# Supplementary material for: Prevalence and determinants of maternal psychological distress: A hospital- based cross-sectional study among pregnant women attending antenatal care in Dar-es-salaam, Tanzania
Source: PLOS Ment Health. 2025 Oct 1;2(10):e0000315. doi: 10.1371/journal.pmen.0000315 (PMC12798589; doi:10.1371/journal.pmen.0000315)
Supplement: Attachment — Submitted filename: Response to Reviewers.docx [file pmen.0000315.s003.docx]

**A Rebuttal Letter**

Dear Editor and Reviewers,

We sincerely thank you for your thoughtful and constructive comments on our manuscript. We appreciate the time and expertise you dedicated to reviewing this work. Below, is the detailed point- by-point responses.

Reviewer comments are presented in **bold and *Italicized,*** followed below with our responses on each point.

# RESPONSE TO REVIEWERS

**Editor Comments:**

1. ***We noticed you have some minor occurrence of overlapping text with the following previous publication(s), which needs to be addressed:***

***- https://doi.org/10.21203/rs.3.rs-3287792/v1***

***- DOI:10.1186/1472-698X-13-4***

***-*** [***https://doi.org/10.3389/fpsyg.2021.627604***](https://doi.org/10.3389/fpsyg.2021.627604)

Response: We have reviewed the manuscript for overlap, rephrased overlapped text from the abstract, introduction and method section and tool descriptions outside the methodology, and ensured all sources are properly cited.

2. ***In your revision ensure you cite all your sources (including your own works), and quote or rephrase any duplicated text outside the methods section. Further consideration is dependent on these concerns being addressed.***

Response: Thank you, we ensured that all sources have been cited correctly and rephrased identified duplicated text. We have also reviewed our entire reference list carefully and confirm that none of the cited papers have been retracted.

We would also like to mentioned that based on the raised valid comments from reviewer **#1** and **#2** we have added several new references to the introduction as the need to provide global prevalence and regional data concerning psychological distress among women in normal population, as well as at the methods section, on studies in Tanzania which have used similar Intimate Partner violence tool. We updated the reference list and adjusted the numbering throughout the manuscript accordingly.

**RESPONSE TO REVIEWERS**

**Response to Reviewer # 1**

Comment on Abstract: ***You may remove the subheadings from abstract and put it into one single paragraph***

Response: Thank you for this helpful suggestion. We have revised the Abstract, removed subheadings and presented it as one continuous paragraph to enhance flow as recommended

**Comment on Introduction Section**: ***On 1st Paragraph, it is better to begin introduction with a generalized term rather than defining “Psychological Distress” For example, I can suggest you to begin with terms like, Mental health/Emotional challenges during pregnancy. Then you can define PD. It will help the non-professional readers and students to understand the vast meaning of 'Psychological***

***Distress'.”***

Response: We appreciate this valuable recommendation. We have restructured the opening paragraph on the Introduction to begin with a broader statement facilitating an understanding on how emotional challenges during pregnancy influence wellbeing, before introducing the definition of psychological distress.

***2nd Paragraph: Kindly include global prevalence of PD among women overall and then highlight the prevalence among pregnant women to differentiate the sensitivity of mental health at this period***

Response: Thank you for pointing this out. We have added recent global estimates of psychological distress among women of reproductive age, using updated data from the Global Burden of Disease 2019 study, and highlighted the higher prevalence among pregnant women specifically, to demonstrate the unique mental health burden during this period.

***3^rd^ Paragraph: Add information regarding contributing factors influencing distress among pregnant women in Tanzania to strengthen the justification. If there’s no data in Tanzania related to this, you can look for data from neighboring or low- and middle- income countries around Tanzania so that the justification gets more highlight by mentioning the study gap from those countries.***

*Response: We acknowledged that it was important to strengthen the justification on contributing factors of psychological distress in pregnancy with relevance to our contexts, therefore we have expanded this section by adding relevant Tanzanian studies (Katiti et al., 2016), regional studies from Kenya (Mutiso et al., 2018) and Uganda (Nakku et al., 2016), that demonstrate key contributing factors.*

**Comment on Result*: Please mention the number of any missing or incomplete data while cleaning or how many participants dropped out of the study (if any. Finally, PLOS encourages authors to reflect on why the findings matter, particularly for Tanzanians. You can briefly mention in the conclusion about potential policy relevance in Tanzania or similar LMIC contexts (1-2 sentences).***

Response: Thank you for raising this important concern. We have clarified in the Results section that; all 287 participants completed the study and no data were missing or incomplete. We have also added sentences to the Conclusion section that highlight the potential policy implications of our findings for

Tanzania’s maternal health programs, and for similar low- and middle-income countries seeking to strengthen mental health integration into antenatal care.

**Response to Reviewer #2**

***MAJOR ISSUES:***

1. ***While the results of the study are valid, the study's title does not seem to capture the fact that the study is specifically about the prevalence and determinants of psychological distress among pregnant women. It will be helpful to the audience to ensure that the title reflects these two concepts***

Response: Thank you for this valuable observation. We have revised the manuscript title to clearly reflect both the prevalence and determinants aspects of the study. The new title is: Prevalence and Determinants of Maternal Psychological Distress: A Hospital-based Cross-Sectional Study among Pregnant Women Attending Antenatal Care in Dar-es-salaam, Tanzania.

MINOR ISSUES:

1. ***Under 'methodology', specifically the paragraph on demographic information, it will be good to provide references to the studies that formed the basis for structuring the demography aspect of the questionnaire.***

Response: We appreciate this suggestion. We have provided references based on regional studies that informed the selection and structure of the sociodemographic and clinical characteristics in our questionnaire (Mahenge et al., 2015; Tesfaye et al., 2023; Spedding et al., 2020)

2. ***Under 'methodology', specifically section on experience of intimate partner violence, it will be helpful to provide references to studies in Tanzania that have validated and/or used the Women’s Health and Domestic Violence against Women tool as you stated in line 4 of that section.***

Response: Thank you for this helpful input. We have strengthened the methodology section on “experience of Intimate Partner violence” assessment by adding additional Tanzanian studies that have used similar tool of, WHO Multi-Country Study tool for assessing it. These include Magnusson et al. (2021), Manongi et al. (2020), Mahenge et al. (2018), and Katiti et al. (2016), and since these were new added references, we updated the reference list accordingly.

3. ***Still under the section on experience of intimate partner violence, can you explain whether reducing the assessment period from 12 months to the current pregnancy (considering that for some women it might be as short as 3 months, etc.) does not significantly affect the tools validity? How sure are we of this?***

Response***:*** This is such a thoughtful question we appreciate for raising it up. We have additionally clarified in the methodology section, that the recall period was shortened from 12 months to the

current pregnancy period, to improve contextual relevance and reduce recall bias. We further justify this by referencing previous Tanzanian studies that have used similar time adjustment, demonstrating that IPV during pregnancy can be reliably captured during current pregnancy (Mahenge et al., 2016; Katiti et al., 2016).

4. ***In paragraph 5 of the discussion, you stated:***

***"Looking at unintended pregnancy and its lack of association, a meta-analysis of studies conducted in low and middle-income countries, reports that women with unplanned pregnancy tend to delay beginning their antenatal care (26,27). While this cannot be surmised from the study findings, it is notable that majority of the participants were in their second and third trimesters, and that the study was unable to capture participants in their early stages of pregnancy."***

***Could you clarify how the findings of the studies you cited explain your finding of a lack of association between unintended pregnancy and psychological distress? It is not so clear from the paragraph.***

Response: Thank you, we acknowledge that this section needed clarification, so we have clarified this part of the discussion and explain better why unintended pregnancy showed no significant association with psychological distress in our findings. We have now clearly outlined how delayed antenatal care and psychological adjustment may have contributed to this, and we have also emphasized that marital status and partner support may have buffered the emotional impact of unintended pregnancy in consistent with recent literature we have cited.

**Conclusion**

Again, we express our appreciations to the Editor, and the Reviewers for your valuable feedback that has greatly strengthened the clarity, and contextual relevance of our manuscript. We appreciate the opportunity to revise our work and hope that our responses and changes meet the required standards.

We look forward to your further consideration of this revised submission. Thank you.

With kind regards, Glory Cuthbert
