# Supplementary material for: Prevalence and determinants of maternal psychological distress: A hospital- based cross-sectional study among pregnant women attending antenatal care in Dar-es-salaam, Tanzania
Source: PLOS Ment Health. 2025 Oct 1;2(10):e0000315. doi: 10.1371/journal.pmen.0000315 (PMC12798589; doi:10.1371/journal.pmen.0000315)
Supplement: Attachment — Submitted filename: Response to Reviewers.R2.docx [file pmen.0000315.s004.docx]

**A Rebuttal Letter**

Dear Editor and Reviewers,

We thank you for your comments on our manuscript. We appreciate the time and expertise you dedicated to reviewing this work, below are responses.

Reviewer’s comments are presented in **bold and *Italicized,*** followed below with our responses on each point.

**RESPONSE TO REVIEWERS**

**RESPONSE TO REVIEWERS**

**Response to Reviewer # 3**

1. Comment on Objectives: ***The research objectives should be stated equally in the abstract, introduction, and discussion***

Response: Thank you for this important observation. We have now revised the objectives across all sections to ensure consistency as pointed out. This change has been made in the Abstract, Introduction, and Discussion sections.

2. Comment on the Abstract**: *What do you mean by key factors? This is vague. It would be better if key factors were specifically named****.*

Response: Thank you, we meant associated factors when we used the term “key factors. To maintain clarity of the work, we have now replaced it with “associated factors”

3.Comment on the Introduction*:* ***All abbreviations (ANC) (MNH) should be written out in full the first time they appear in the text.***

Response: Thank you for pointing this out, the abbreviations are now written in full in first time mention.

4.Comment on the tools:

A: ***Details of validity and reliability of the validated version of the Kessler Psychological Distress Scale (K-10) in Tanzania***.

Response: Thank you, we have added mor details on the validity and reliability of K-10 in Tanzania

B: ***Please, write the details of the OSSS-3 and IPV tools***

Response: Thank you, we have expanded this section and added mor information as suggested

**Response to Reviewer 4**

1.Comment on ethical consideration: ***What was the ethical approach for "disposing" the participants, if found with significant mental health issue?***

Response: Thank you for highlighting this. We have now clarified more on the ethical handling of participants in Ethical consideration under the Methods section.

2.Comment on Outcome**: *What impact did you notice on the birth outcome (ultimate outcome of interest)***

Response: Thank you so much for this thoughtful question. The scope of our study was limited to assessing the prevalence and associated factors of psychological distress among pregnant women during antenatal care. As such, it did not include follow-up to measure birth outcomes. We appreciate the importance of this line of inquiry and suggest it as a valuable focus for future longitudinal research in maternal mental health in Tanzania

**Closure**

We express our appreciations to the Editor, and the Reviewers for your continuous feedback. We appreciate the opportunity to revise our work and hope that our responses and changes meet the required standards.

We look forward to your further consideration of this revised submission. Thank you.

With kind regards,

Glory Cuthbert
