# Supplementary material for: Prevalence and determinants of maternal psychological distress: A hospital- based cross-sectional study among pregnant women attending antenatal care in Dar-es-salaam, Tanzania
Source: PLOS Ment Health. 2025 Oct 1;2(10):e0000315. doi: 10.1371/journal.pmen.0000315 (PMC12798589; doi:10.1371/journal.pmen.0000315)
Supplement: Attachment — Submitted filename: Response to Reviewers.R3.docx [file pmen.0000315.s005.docx]

**A Rebuttal Letter**

Dear Editor,

We thank you for your ongoing valuable feedback on our manuscript. We appreciate the efforts you’ve been dedicating to review this work, kindly find the response below.

Editor’s comments are presented in **bold and *Italicized,*** followed below with our response.

**RESPONSE TO THE EDITOR**

1. Comment on Data Analysis: ***Odd Ratios are used for rare events (less than 10%). I can see that your prevalence is 31.2%. I advise that you use prevalence rate (PR) instead of Odd ratio (OR). So, COR will be replaced with cPR and AOR replaced with aPR.***

Response:

Thank you so much Editor, for this important guidance. We acknowledge this valuable input and I have careful rerun our data using Poisson regression with robust variance, which is appropriate for non-rare outcomes. This has enabled us to replace odds ratios (cOR/aOR) with crude and adjusted prevalence ratios (cPR and aPR), as importantly advised.

We have updated the respective information at the Abstract, Methods, Results, Tables 2, Results, and Discussion to align with the slight changes in the analysis. All these changes have been clearly highlighted in the revised manuscript document.

**Closure**

Again, we express our sincere appreciations to for your continuous feedback. We look forward to your further consideration of this revised submission.

Thank you.

With kind regards,

Glory Cuthbert
